# A dynamically minimalist cognitive explanation of musical preference: is familiarity everything?

**DOI:** 10.3389/fpsyg.2014.00038

**Published:** 2014-02-06

**Authors:** Emery Schubert, David J. Hargreaves, Adrian C. North

**Affiliations:** ^1^Empirical Musicology Group, School of the Arts and Media, University of New South WalesSydney, NSW, Australia; ^2^Applied Music Research Centre, Roehampton UniversityRoehampton Lane, London, UK; ^3^School of Psychology and Speech Pathology, Curtin UniversityPerth, WA, Australia

**Keywords:** musical experience, cognitive model, reciprocal-feedback model, spreading activation, mind-body, neural networks, preference, familiarity

## Abstract

This paper examines the idea that attraction to music is generated at a cognitive level through the formation and activation of networks of interlinked “nodes.” Although the networks involved are vast, the basic mechanism for activating the links is relatively simple. Two comprehensive cognitive-behavioral models of musical engagement are examined with the aim of identifying the underlying cognitive mechanisms and processes involved in musical experience. A “dynamical minimalism” approach (after Nowak, 2004) is applied to re-interpret musical engagement (listening, performing, composing, or imagining any of these) and to revise the latest version of the reciprocal-feedback model (RFM) of music processing. Specifically, a single cognitive mechanism of “spreading activation” through previously associated networks is proposed as a pleasurable outcome of musical engagement. This mechanism underlies the dynamic interaction of the various components of the RFM, and can thereby explain the generation of positive affects in the listener’s musical experience. This includes determinants of that experience stemming from the characteristics of the individual engaging in the musical activity (whether listener, composer, improviser, or performer), the situation and contexts (e.g., social factors), and the music (e.g., genre, structural features). The theory calls for new directions for future research, two being (1) further investigation of the components of the RFM to better understand musical experience and (2) more rigorous scrutiny of common findings about the salience of familiarity in musical experience and preference.

This paper presents a cognitive model that explains musical experience. In particular, we seek a parsimonious account of the many factors (or determinants) that contribute to musical preference and pleasure. To this end some cognitive models of musical experience are revisited with a view to generating testable hypotheses. After reviewing two theoretical models from music psychology that will be integrated into a “cognitive-behavioral model of musical experience,” a single underlying cognitive mechanism is proposed that dynamically interacts with the various components of the model to provide a simple but enriched theoretical understanding of musical experience. We examine the empirical data that the model explains, discuss some of its weaknesses, and describe how it can be further tested.

## COGNITIVE-BEHAVIORAL MODELS

Models of esthetic musical experience have tried to explain basic, fundamental responses to music, such as preference and taste (for reviews, see Finnäs, 1989; North and Hargreaves, 1996; Hargreaves and North, 2010). Preference in particular was considered suitable for early, behaviorist studies because a simple dependent variable measure could be employed, such as a scale of liking, with a range of potential independent variables that could be used as predictors (e.g., gender, age, and various musical characteristics). Although the term “musical taste” has been used to mean a variety of things, including preference (e.g., Schuessler, 1948) modern definitions of musical taste recognize that the concept reflects the “overall patterning of an individual’s preferences” (Hargreaves and North, 2010, p. 517), and so it is used to refer to long term, broader likes and dislikes, such as particular musical styles, as distinct from a short-term liking for a particular song or piece relative to another. LeBlanc used both the terms preference and taste in developing his model (LeBlanc, 1980, 1982; LeBlanc et al., 1999), which consisted of a comprehensive set of components to explain musical preference at a given point in the listening experience. The model attempted to account for several important findings, while acknowledging the multifaceted complexity and filtering processes that may be involved in such a preference decision.

Regarding predictors of the kind of music people liked, age, and gender were considered among the most important at that time. For example, females were found to have broader tastes in music than males, and younger listeners exhibited greater “open-earedness” (after Hargreaves, 1982) than older listeners. These two components (age, or “maturation,” and sex) were part of a total of 13 components that contributed to the “Listener” variables of the model – the background factors of the individual that contribute to preference. LeBlanc’s model was hierarchical, with the “music” (e.g., its physical properties) and the “cultural environment” (referring to who else is present or influencing the listening experience – friends, authority figures, etc.) variables each occupying the bottom level of the hierarchy. The “Listener” variables are sandwiched between these two sets of variables (below) and preference decision related components (above).

Like LeBlanc, many researchers argue that variables and components such as these are interactive (Finnäs, 1989; Hargreaves et al., 2005). That is, although LeBlanc proposed a hierarchy, each level in the hierarchy had a link (arrow) that allowed one variable in the model to be influenced by others regardless of its hierarchical position. In their reciprocal-feedback model (RFM), Hargreaves et al. (2005) made explicit in its title the interactivity of the determinants of musical response, and reduced reliance on any hierarchical order of processing. Furthermore, their model intended to explain musical communication, which included musical preference responses. In its early form, the RFM consisted of four boxes, three being the determinants of the central “response” box, which were labeled “Music,” “Listener,” and “Situation and Contexts.” Each of the boxes was linked to each of the others. While there are some parallels with LeBlanc’s model, the reciprocal-feedback format integrated new findings about what was then known regarding music preference and communication research. For example, while social interaction and esthetic emotion are at the periphery of LeBlanc’s layout, emotion and social context were amongst the contents of the Responses and the Situations and Contexts boxes respectively, reflecting the view that emotions and the social context play an important role in preference judgments, in accord with contemporary research on these factors (Hargreaves and North, 1997; Juslin and Sloboda, 2001).

Research carried out after the publication of LeBlanc’s model cemented the important role of emotion in the enjoyment of music (Panksepp, 1995; Rickard, 2004; Schubert, 2007a). Perhaps most importantly, as far as the present enquiry is concerned, the RFM does not necessitate that preference is an output that results from a hierarchy of inputs and filters. The response box includes a range of “outputs,” including affective, physiological, and cognitive components, that may or may not be influenced by a variety of determinants.

The RFM underwent additional refinements, including the proposition of a “performance” model which ran in parallel with the response model, and the further proposition that the two models in combination might be used to explain musical communication. The most recent development of the RFM (Hargreaves et al., 2012) involved the synthesis of the “response” and “performance” models into a single model in which the central component of the central box is specified as “Imagination,” which is manifested in two main ways, namely in production and perception. Perception refers to the components of the responses box in the earlier version of the RFM, and production refers to all the components involved in the expressive and motor outputs of the performer/composer, and indeed in musical creativity. The most radical component of the revised model is the inclusion of the imagination factor. Imagination is presented as the central core of the model because it represents the cognitive processes underlying musical experience. Whereas the original model was intended to explain musical response, performance, and communication, placing imagination at the core of the model allows for the integration of all of the creative acts involved in music making, and also changes the nature of the model such that it now deals in essence with the mental activity involved in music processing. Indeed, it will be argued here that the revised model provides an apt explanation that covers much of the sum total of musical experience.

## REDUCTION OF THE PROBLEM

The models described above represent musical experience as a collection of categorical blocks, each of which is able to mutually interact with the others. They reflect the evidence at the time at which the research was available, and have historical origins in the philosophical position referred to as reductionism – the separable components responsible for the emergent phenomenon under investigation (in this case, musical experience). While this leaves us with an understanding of musical experiences that is iteratively deeper, it also raises the question of how to manage the increasing complexity of such a model. Nowak (2004) argued that it may be possible to find a compromise between incomprehensible complexity with richness of understanding, versus simplicity and triviality. He proposed a system of theory development referred to as “dynamical minimalism” in which the explanations of the behavior of systems can be understood as evolving in time through repeated interaction of simple mechanisms.

As mentioned, Hargreaves (2012) proposed that imagination (and its components) is at the core of the revised RFM, and recent developments in cognitive psychology suggest that the concept of imagination may be the place to find a simple mechanism. In the present model, imagination needs to be understood in two ways: first, it refers to the self-reportable, experiential level of fantasy, make-believe, remembering, planning, and so forth, in the absence of physically sensed stimuli (sight, sound, etc.). Second, it can be understood at a cognitive level as involving different networks of association that are a part of mental processing. Those networks function as a large, distributed set of nodes that are responsible for the formation^1^, storage and retrieval of memories, both motor and perceptual (Fuster, 1997), but equally important, they are involved in the formation of novel associations and activities. This second concept of imagination, particularly through its interaction with the first, experiential concept of imagination, provides a parsimonious explanation not only of responses to music, but also of the creation of music as part of the production (e.g., playing, composing, improvising) process.

Hargreaves (2012) indicates the utility of the network approach by identifying three types: musical, social-cultural, and “personal” networks, and these align with Folkestad’s 2012 conception that all music heard is stored in the mind of the listener in what he calls a “personal inner music library.” Social-cultural networks may be thought of as determining the ways in which musical interactions and experiences are shaped by cultural norms and contexts, such as hearing traditional Indian music when eating at an Indian restaurant (North et al., 2004; Yeoh and North, 2009). Personal networks are the most individualized networks that map out the various experiences and associations of an individual: they do so by combining aspects of musical and social-cultural networks. Thus, imagination can be seen as operating through a collection of interacting cognitive networks.

A criticism of this version of the model is that, as a result of the inclusion of imagination, the theory traverses different levels of explanation (e.g., see Carlston, 2010). The original version of the model was largely phenomenological, taken here to mean behavioral (e.g., self-reporting the liking a piece of music) and observable (age, gender, personality traits assessed through a psychometric instrument, the characteristics of a piece of music, and so on). Imagination as conceived here, however, consists of the internal workings of the mind that are concerned with musical experiences which may not always be directly accessible through behavior and observation, and may even be contrary to the observations and behaviors of the individual having them. In response to such a criticism, Hackman (2003) argued that examination of different levels of explanation can lead to more robust understanding and “findings that otherwise would have escaped notice” (p. 910), making the addition of the imagination factor even more significant for the purpose of theory development.

Our intention here is accordingly to identify a minimum number of underlying cognitive mechanisms that help explain the behavioral and observable data, and to identify and justify that which possesses the most explanatory power.

## SPREADING ACTIVATION

We suggest that the single, best available contender for explaining musical experience and esthetic pleasure is spreading activation theory (e.g., Schubert, 1996, 2009–2010, 2012). Spreading activation refers directly to the mental processing portion of the RFM in that it depends on a mental architecture consisting of a vast network of nodes (as does the imagination factor of the RFM). When a perception or action occurs, specialized networks representing that perception/action process are activated through the connections of that network. For example, the act of walking requires neural networks that prepare for and execute the act by sending motor instructions including bodily co-ordination (Cruse et al., 1995). The instructions are not explicit, but are distributed through the network as a result of learning and maturation (Collins and Loftus, 1975; Smith and Queller, 2004).

The principles of spreading activation can be found in early English speaking psychological writings through the work of William James, and in particular his elementary law of association: “When two elementary brain-processes have been active together or in succession, one of them, on reoccurring, tends to propagate its excitement into the other” (James, 1890/1950, p. 566). In the case of perception, a familiar visual stimulus will activate one set of networks, and if that visual stimulus is of a musician, for example, another set of networks may be activated as a result of the music that the musician is playing. The mental representations of the music and the visual stimulus (of the musician) are combined to form another, integrated network (e.g., Anderson, 1988; Fuster, 1997; Fuster et al., 2000; Laughlin and Sejnowski, 2003). These combinations of networks are linked together by the appropriation of a new network (if the connection has not previously been made). These representations and associations in some psychological models are referred to as long-term memory (Sweatt, 2003). The later re-activation of a part of the network [e.g., the sight (perception) of the musician] can activate the associated portions (e.g., the music that the musician previously played) even in the physical absence of that additional stimulus. Each of the concepts that the different networks represent – whether it be the piece of music, the components of the music, the musician, the environment of the music – are called “nodes” (Anderson, 1983). Total activation is therefore determined by the combination of quasi-digital transmission of signals via nodes. The nodes transmit or they do not – on or off. It is the sum of the transmitting node outputs that form overall activation and, in effect, produces the intensity of the arousal.

Martindale (1984, 1988) proposed a simple mechanism that explained hedonic preference in terms of these interconnected nodes, namely that the process of activation of nodes is in itself pleasurable, provided that the listener is in a disinterested state. The theory specifically addresses the circumstance of esthetic experience (such as playing and/or listening to music), which was adopted and modified by Schubert (1996, 2009–2010) and which led to the development of a spreading activation theory of esthetic and creative experience (Schubert, 2012). An important point in the model that we are proposing, and in that of Martindale, is that the basic tenet of the model requires that the listener be in a state of esthetic contemplation, which we will refer to as an esthetic context (e.g., Frijda, 1989). Thus, listening to “music” in a dangerous environment, or day-to-day sounds (such as the ring tone of a mobile phone, or jack-hammer at a construction site) is not here considered as being in an esthetic context (for further discussion, see Schubert, 2009–2010).

## EXPLAINING MUSICAL EXPERIENCE THROUGH A DYNAMICALLY MINIMAL MODEL OF SPREADING ACTIVATION

It is possible to build an explanatory sequence based on the assumption of a simple mechanism that drives esthetic (in this case musical) experience. The spreading activation thesis predicts, within an esthetic context (1) that a mental representation (node) must be activated in order to generate esthetic pleasure, which implies that; (2) a representation must be present, meaning that: (3) the mental representation must first have been “formed.” These three principles can be satisfied in various ways. Formation of mental representation is the basis of learning and experience. For example, a mental representation can be formed, without conscious attention, by mere exposure (Zajonc, 1968; Monahan et al., 2000). Mere exposure and any other driver of mental representation formation can be translated into the phenomenological world as familiarity. Familiarity – a collative variable in the RFM (Berlyne, 1970) – can therefore be explained through the presence of mental representation of a piece of music, a style of music, a performer, and so on. The activation of that mental representation is pleasurable, and is reflected in numerous studies (see reviews of music preference cited above, as well as Gaver and Mandler, 1987; Schubert, 2007a; Pereira et al., 2011).

Studies in the social psychology of music have presented an increasingly sophisticated understanding of musical experience. Using spreading activation as the underlying mechanistic driver of musical experience, social context may be viewed as a facilitator or inhibitor of musical exposure. Being with a friend, having a role model, or wanting to be part of the in-group (Tarrant et al., 2001; Pitts, 2002) will influence the quantity and type of music to which one is exposed, but the music will also form associations with the context and social connections that are experienced during the music listening experiences. When playing music at a campfire with friends for the first time, the network of associations with the environment (the campfire and atmosphere), the friends and the music will form new networks which represent the co-occurrence of the music and social context, and thus future experiences involving any or all of these components can lead to a large amount of activation spreading through the network at a subsequent activation involving any or all of those components (the campfire, the friends and/or the music). One event (e.g., the music) may trigger – activate – another (e.g., memory of being at the camp). A positive memory may produce a sense of awe, pleasure or frisson (Lowis, 1998). Our argument is that spreading activation underlies these affective responses: it explains why situations and contexts are such an important part of the musical experience, and suggests that the external influence of context, while critically important, can at the same time be explained mechanistically.

The social connections that influence an individual’s musical experience (friends, influential people, etc.) have relevance also for another concept that has received considerable recent attention, namely that of emotional contagion and empathy (Sawyer, 2006; Juslin and Västfjäll, 2008; Woody and McPherson, 2010). When we are with people we like we tend to adopt their mood or emotional state (Hatfield et al., 2009). We are happy to hear of a friend’s good news, and feel sad when she/he has been through a difficult time (Cialdini et al., 1997). This “capturing” of mood is referred to as emotional contagion, and when the individual is showing involved concern with that person we refer to the experience as empathy (Watt, 2005). In neuroscientific research a picture is beginning to emerge that such empathic experiences are produced by activation of, among other things, mirror circuits (Preston and De Waal, 2002; Platek et al., 2005; Schulte-Rüther et al., 2007; De Waal, 2008; Walter, 2012; Schubert, 2013). The assertion that mirror circuits are the mechanism of empathy is not without controversy (Decety, 2010), although the presence of a specialist circuit for processing our strong sensitivity to interhuman interaction is plausible, if not biologically critical (e.g., see Walter, 2012). Connection with others – as a cause or result of empathy – has an important role to play in musical experience, too (Woody and McPherson, 2010). These social interactions lead to activation of relevant networks representing social engagement. The key point is that the linking of these cognitive networks with (pieces of) music, through the principle of spreading activation (which is pleasurable in an esthetic context) provides a mechanistic explanation of the context determinants of the RFM. Although some recent work on contagion comes from neuroscientific research (e.g., Gallese, 2003; Singer and Lamm, 2009; Decety, 2010), the current account employs an explicitly cognitive framework.

Returning to our camp fire example, the spreading activation account proposes that the social relations among the individuals present are activating a large number of contagion/mirror circuits concomitantly, and that the connections between circuits these and the music create even larger amounts of activation. In short, it may be that social interaction is an evolutionarily important, convenient way of activating many nodes, and musical activation links these experiences together, allowing later listening to that music to re-activate networks of memories and feelings (Chartrand and Dalton, 2009).

**FIGURE 1:**
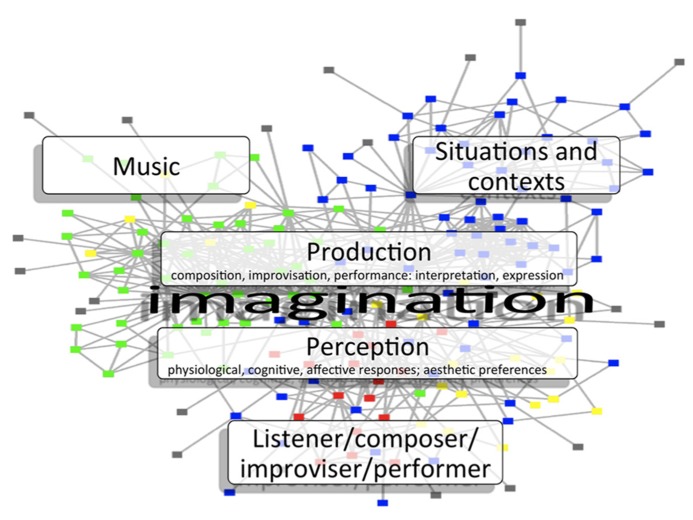
**Schematic representation of spreading activation through the components of the reciprocal-feedback model (RFM; based on Hargreaves, 2012).** The arrows connecting the determinant boxes in previous versions of the RFM have been replaced here by networks of nodes, shown in schematic form in the background, and representing a cognitive level of explanation. The networks form mental representations of perceptions, actions, emotions, and thoughts, including those pertaining to the three determinants of musical experience: music, situations, and contexts, and Person (Listener/composer/improviser/performer). Activation of one part of the network spreads activation to other previously associated networks. The central role of imagination is represented schematically in the diagram in terms of musical experience (foreground), and of internal, mental representation (background).

The spreading activation mechanism fulfils the criterion of being minimal because it is a single, important principle that dynamically interacts with the various components of musical experience – the more activation, the more pleasure. We provide a schematic representation that shows how the mechanism unifies the components of the RFM in **Figure 1**. Such an approach is easy to criticize because it explains a great deal with very little, and some of the criticisms will be addressed in the following section.

## PREDICTIONS AND CRITICISMS

The current thesis proposes that the spreading activation mechanism presents both a parsimonious explanation of an extremely complex set of phenomena, and provides some testable hypotheses that may lead to further modifications or rejection.

In terms of prediction, spreading activation implies that a mental representation must be present before activation can begin. The self-reported, introspective, phenomenological experience of a mental representation will, in its simplest form, be the sensation of recognition, and will therefore often be connected with familiarity (Gabrieli, 1998; Jeneson et al., 2010). In this respect, a naïve interpretation of the spreading activation mechanism suggests that familiarity will be the most important predictor of esthetic experience, and in particular, of preference. While there is much to say about this prediction, a few advance comments about prototype theory are required.

As with spreading activation, the theory of prototypicality predicts that people will like the most prototypical music that they hear: that is, music that sounds most similar to their existing mental representations of musical styles and pieces. Prototypicality theory was championed by Martindale and colleagues (Martindale and Moore, 1988, 1989; Martindale et al., 1990) but fell out of favor in the late 1990s for several reasons. One important reason was discussed by North and Hargreaves (2000), who argued that prototypicality may have found support in the first place because of the stimuli used to examine the theory. The importance of prototypicality, they argued, was proportional to the extent to which the stimuli in question varied in their prototypicality. Spreading activation theory (which, incidentally, was inspired by the work of Martindale – who had in place many of the principles of the present theory) does not imply any bias as to which component of the RFM will be a better predictor of esthetic preference. Familiarity is one, and only one conscious manifestation of pre-existing mental representations, and explains prototypicality too, because prototypicality is the result of networks representing one stimulus (a novel piece of music) activating all “adjacent” networks that share features with the novel piece, should they exist. Familiarity is not an exclusive factor in making predictions about liking, as it intrinsically involves other factors. Our proposed theory consequently departs from Martindale’s detailed focus on prototypicality.

Furthermore, our theory draws attention to the way that preference for musical style interacts with preference for individual musical pieces, which lends itself to easier direct inspection, such as comparisons between music played in two or more different styles and contexts. The context that activates the greater number of mental representations – for example through exposure and cultural norms, will be more liked. Evidence of this prediction can be found in a study by North and Hargreaves (1997). In that study, pop songs played in styles that were more familiar to the participants were liked more than when played in a less familiar style, even if the liked styles were not that of the “original” piece.

One important esthetic principle that spreading activation does not explain is why familiarity can increase without monotonic increase in enjoyment. That is, it is generally accepted in the literature that preference increases with familiarity, but at a certain point, when a piece becomes “over” familiar, enjoyment diminishes. This conclusion is encapsulated in the principle of the inverted-U curve (Berlyne, 1970; Heyduk, 1975; Hargreaves, 1984; Schubert, 2010), and was first identified by the early German psychologists, particularly through the work of Wundt (1905). To retain the spreading activation mechanism explanation, an additional mechanism could be added, as proposed by Martindale, such that an “activation threshold” that dynamically alters how much activation is required before a node (representing a network) can be activated. That is, massed exposure to an esthetic stimulus will lead, in general, to an increase in the activation threshold, eventually stopping the node (the one representing the stimulus that is being presented) from activating. The consequent blocking reduces the amount of activation that can spread through the network (for more details, see Martindale, 1984; Schubert, 1996; Schellenberg et al., 2012). However, our aim is deliberately to set a research agenda that identifies the *single* best explanatory mechanism for esthetic experience, without excluding additional mechanisms from explaining musical experience and preference.

There is a second related issue concerning the notion of familiarity, which also requires detailed exposition. Put simply, our description so far suggests that exposure, familiarity, and spreading activation are similar concepts. However, the notion of conscious attention means that it seems sensible to differentiate these terms (Bargh and Ferguson, 2000; Hubbard, 2007). Conscious attention directs the nature of the representation formed (or activated) so that anything in consciousness at the time influences the representation that is formed (or activated). For instance, if one hears a piece of music and is told that it represents a given musical style, then this information will become part of the representation of that piece. In this context, exposure can be equated with the perceptual mechanism of having heard the music, whereas familiarity implies the forming and repeated activation of a particular node (or set of nodes), which seems analogous to the concept of consciousness: it can be argued therefore that familiarity equates to “exposure plus consciousness,” and that spreading activation is the process by which consciousness shapes exposure into familiar music that is cognitively meaningful.

A clear prediction follows from this, namely that the greater the degree of conscious effort devoted to active processing of a given exposure to music so, it would be assumed, the greater the extent and richness of the nodes activated. In contrast, mere exposure to music with little or no ensuing conscious effort (i.e., spreading activation) would lead to relatively little activation of a limited number of nodes and an impoverished pattern of activation.

However, mere exposure still has an important role to play in mental representation, and this can be further exemplified in the importance of repeated material used within a piece of music (Ockelford, 2005). Within-piece repetition has the ability to engage the listener’s attention and emotional response (Livingstone et al., 2011; Margulis, 2013a,b) as well as with-in piece repetition with elaboration and ornamentation, such as the Theme and Variations form (Keller and Schubert, 2011) and the “hook” in popular music (Traut, 2005). This furthers the present argument that the search for existing mental representations, which can be of a musical fragment, will lead to a positive affective outcome for the listener.

Our theory, therefore, draws together disparate, significant explanations in cognitive musical organization (Deliege, 2001a), by proposing that terminology used to indicate familiar musical fragments, a particular musical style (North and Hargreaves, 1997; Deliege, 2001b), a musical schema (Bharucha, 1987; Justus and Bharucha, 2002) and prototypically (op. cit.) all point to mental representations that are activated by music factors specified within the RFM (which here includes performing and imagining). This is in addition to the veridical activation of linked mental representations that will occur when listening to a familiar piece of music.

Perhaps the major criticism of spreading activation theory is that it explains too much, and therefore lacks predictive utility. Our response to this criticism is that it predicts that what is important in musical experience (that is, in an esthetic context) is the richness of networks that the music activates, whether this be other music of a similar style (such as prototypicality), memories of past events (“evaluative condition” – see for example, Juslin and Västfjäll, 2008), contextual information, and so on. No single theory has been cited that predicts that the “richest” musical experiences will be acquired by the maximization of the spread of activation to different cognitive networks within and across the various components of the RFM. Interestingly, William James, again, showed premonitions of the notion of “spreading activation” but with the missing assertion that this is a pleasurable activity when it occurs in an esthetic context:

The amount of activity at any given point in the brain-cortex is the sum of the tendencies of all other points to discharge into it, such tendencies being proportionate (1) to the number of times the excitement of each other point may have accompanied that of the point in question; (2) to the intensity of such excitements; and (3) to the absence of any rival point functionally disconnected with the first point, into which the discharges might be diverted (p. 567).

Our dynamical minimalism approach has four advantages over other theories of musical experience, namely that: (1) It crystallizes the need to distinguish between the various components of musical experiences (complexity, prototypicality, but also personality, social and contextual factors and so forth), which are brought into focus by the RFM; (2) It predicts that in an esthetic context any of these components can contribute to positive esthetic experiences; (3) While other cognitive theories of musical preference lay out the various determinants of musical preference, they rarely identify the reason for the generation of preference or pleasure: our theory explicitly identifies the causal mechanism of musical (esthetic) pleasure – spreading activation, and (4) Our theory sets a research agenda that requires a focus on the relative contribution of each component of the RFM to the overall experience.

## CONCLUSION

The single cognitive mechanism of spreading activation provides a potential solution to the problem of achieving dynamical minimalism in theories of musical experience. Spreading activation, we have proposed, is a basic mechanism that interacts with, is shaped by, and forms the various components of the RFM over time. This provides some obvious insights into the nature of musical preference and experience. It predicts that various components of the RFM (musical determinants, cultural and social factors, and listener characteristics) are each contributors to the esthetic experience, and therefore that research should focus on balanced comparisons of the various components and their proportional contribution to the esthetic/musical experience. Of all the determinants, current evidence suggests that familiarity or exposure is the “driving” principle of musical experience (see also Schubert, 2007a; Witvliet and Vrana, 2007; Pereira et al., 2011). Consider, for example, the findings reported at the time of the LeBlanc model related to gender – that young adolescent females have a more positive attitude to music than their male counterparts (Hargreaves et al., 1995). If a cultural norm encourages females to listen to more musical styles than males (e.g., males may be conditioned to believe that some music is less “cool” or less palatable than other music, and therefore avoid it: Tarrant et al., 2001), it is still the differential amounts of exposure, and therefore exposure and familiarity, that underlies the development of preference, not gender differences *per se*.

But the current model clarifies the idea that familiarity and recognition are variables available to the introspection of the individual. Familiarity does not directly identify the cognitive mechanism that underlies the experience. That is, if we understand familiarity as being a result of the “formation” of networks (nodes), we become open not only to how spreading activation can manifest itself in consciousness as familiarity, but that it can also explain the processes of other variables in the RFM. In other words, familiarity is important, but how important is it with respect to other variables and determinants, and how might music psychologists be able to undertake valid comparisons between the effects of two or more of these factors (e.g., familiarity versus prototypicality)? The present thesis does not answer this question, but draws attention to it.

We have therefore attempted to provide a dynamically minimalist explanation of the comprehensive RFM proposed by Hargreaves and colleagues. A simple underlying mechanism – that spreading activation through cognitive networks generates pleasure and other positive affects – dynamically underlies, and is shaped by, the various components of the RFM, and can explain the development of musical experiences over time. That is, the musical *affects* of the components of the RFM can be largely explained by the spreading activation mechanism.

The importance of social context is expressed in terms of the additional amounts of activation the listener experiences when the music is connected with other people, situations and environments, and interrelationships among those. For example, the large amount of activation that can occur when listening to music that a friend likes, or in a social context such as a campfire, may provide a simple, mechanistic explanation of much that is known about the social psychology of music. It may also be the case that the circuits related to empathic behaviors are recruited for activation of a “relationship” with the music itself – between the emotion expressed by the music and the listener’s felt emotion (Gabrielsson, 2002; Dibben, 2004; Kallinen and Ravaja, 2006; Schubert, 2007b; Evans and Schubert, 2008; Schubert, 2013).

Further research will be able to reveal whether this simple cognitive mechanism of spreading activation may help us to understand which of the various components of the RFM account for the largest amount of variance in response and in experience. While reducing the rich and powerful experiences of musical engagement to a simple mechanism may seem overly simplistic to some, from a research perspective, it has the potential to provide stimulation for the generation of many new hypotheses and significant research directions.

## Conflict of Interest Statement

The authors declare that the research was conducted in the absence of any commercial or financial relationships that could be construed as a potential conflict of interest.

## Footnotes

1 In this paper, “formation” is to be thought of as the apprehension or bringing into functionality an otherwise unused cognitive network. It is assumed that vast networks are “physically” present, but serve a function or representation only as a result of experience and maturation.
